# The New Biology as an Example of Newspeak: The Case of Polish Zoology, 1948–1956

**DOI:** 10.1007/s10739-020-09594-6

**Published:** 2020-02-20

**Authors:** Agata Strządała

**Affiliations:** Department of Humanistic Sciences in Medicine, Faculty of Medicine, Wroclaw Medical University, Wybrzeże L. Pasteura 1, 50-367 Wrocław, Poland

**Keywords:** Newspeak, Michurinism, Lysenkoism, New biology, Zoology, Polish biology

## Abstract

The “New Biology” that arose in the Eastern Block during Stalinist times was based on the idea of the heritability of acquired characteristics. In rejecting the paradigm of Mendelian chromosome genetics as well as science-based farming, the New Biology led to a deterioration of scientific life and the free exchange of ideas. In imposing Lysenko’s ideas onto zoology, the New Biology adopted the totalitarian language of Newspeak, which dominated public discourse in communist countries. Newspeak had several defining elements: a limited dictionary, strong valuations, binary oppositions, the magical function of language, militarization, and ritualization of language. In this study, the concept of Newspeak is used to analyze primary sources (publications, speeches, and conference discussions) in Polish zoology in the period between 1948 and 1956. Once the practice of Newspeak began to wane, so did the New Biology that had been founded on this specific ritualistic language.

## Introduction

The adoption of the New Biology in Polish zoology was a consequence of Soviet influence, which after 1945 reached into many areas of political, social, and intellectual life in Poland, including science (Paczkowski 1999; Werblan 2009; Eisler and Kupiecki 1992). The New Biology, based on the idea of heritability of acquired characteristics developed by Soviet agronomist Trofim Lysenko (1898–1976), rejected genetics and science-based farming and agriculture. While in the Soviet Union, the opponents of Lysenko were brutally persecuted, in the Eastern Bloc during Stalinist times (1948–1956),^1^ the enforcement of the New Biology led to the deterioration of scientific life and the free exchange of ideas in many fields of knowledge.

Developments during this eight-year period can be briefly summarized. Although some aspects of Lysenkoism and Michurinism were known in Poland before 1948, their overall reception was very limited in significance and influence. Essentially, they were restricted to the Polish biologist Dembowski (1889–1963) and his closest associates.^2^ Raised in Russia and a graduate of the University of St. Petersburg in 1912, Dembowski’s first attempts to introduce the New Biology in interbellum Poland was a fiasco that slowed down the trajectory of his scientific career. During the Second World War, Dembowski collaborated with the Soviet forces in order to introduce communism into Poland. In 1948, when radicalized communists gained positions of power in Poland, Dembowski continued his career as a politician and a member of the highest circle of power. During the special conference that took place in Warsaw on 30 March 1949, Dembowski proclaimed the New Biology as an official paradigm in Polish science. Henceforth, genetics and any criticism of the New Biology was to be actively and officially confronted.^3^

Although Lysenkoism had been present in public discourse between 1945 and 1948, genetics and other scientific concepts were not openly confronted. From 1948 on, however, the New Biology was officially supported by state authorities and any other non-conforming ideas were publicly denounced.^4^ This change led many prominent scholars, such as Stanisław Skowron, Teodor Marchlewski, and Edmund Malinowski, all experts or at least well-versed in genetics, into a radical, involuntary, and in many ways bogus conversion into practicing the New Biology (Marchlewski 1951b). They had to abandon their areas of expertise, which became politically unacceptable (Strządała 2012a). Some, like Malinowski, were publicly humiliated or stigmatized for using the terms related to genetics. Others, like Włodzimierz Michajłow, turned to the New Biology out of fear of being expelled to the Soviet Union. For many, adopting the New Biology was a form of conformity, while for others it was a form of “protective coloration,” although there were, to be sure, cases of truly dedicated Lysenkoist academicians (Strządała 2013).

Consequently, in the years between 1948 and 1956, the New Biology impacted many aspects of Polish society. School and university programs were modified. Genetics was removed from school textbooks and replaced by the New Biology. University courses in genetics were removed in 1949 and only reintroduced in 1957. Scientific conferences, radio broadcasts, meetings, courses, and publishing campaigns for scientific and popular books and articles were organized to popularize the “new trend” in biology. And outside of the academy, there were attempts to adjust farming and the agricultural sectors to comply with Lysenkoism.

Despite the totalitarian oppression imposed on academia, there was structural opposition if not open resistance. This can be demonstrated by the spurious adoption of the New Biology in which a verbal facade was constructed. Although ostensibly complying with the system, the language employed nonetheless surreptitiously hid and protected some forms of independent thinking. Stalinization of Polish universities was damaging but never fully successful. Polish intelligentsia, shaped by the painful experiences of the partition of Poland and the Second World War, tragically marked by the Nazi occupation, developed an underground culture, including the Secret Teaching Organization (Tajna Organizacja Nauczycielska, or TON),^5^ that later prevented a passive and uncritical reception of Stalinism (Connelly 2013, pp. 218–220). Finally, after 1956, with the political shift and wane of Stalinism in the USSR, the New Biology suffered a rapid decline.

The relationship between Stalinism and science (see Krementsov 1997) and the topic of Lysenkoism in Poland (see de Jong-Lambert 2008; Köhler 2008) have been well described from a number of perspectives. In this article, I will not repeat well-known facts. Rather, I will specifically examine how Lysenkoism was applied and maintained as a linguistic phenomenon. Based on primary sources in the New Biology that appeared in books and articles in Polish journals, as well as records of scientific conferences that took place in Poland or were widely discussed at the time, I analyze the language of the New Biology (“Nowa Biologia”) in Polish zoology from 1948 to 1956.

To deconstruct the terminology used in the publications of the New Biology, I draw on the concept of Newspeak, the theory developed by philologists Michał Głowiński, Petr Fidelius, and Václav Havel that, inspired by George Orwell’s *1984*, describes the official totalitarian language of communist countries (see Young 1991; Tuckerová 2010). The linguistic tropes of the New Biology evidence significant elements identified with Newspeak. As I show, some concepts of the New Biology, which derived from botany, were impossible to apply to the realm of animals. As a result, Lysenkoism in zoology was mainly a verbal ritual rather than a new paradigm in science.

## The Concept of Newspeak and Its Features

The term *Newspeak* was introduced by George Orwell in one of his most famous works, the dystopian novel *1984* (1949). In the book’s Appendix, Orwell described the rules of Newspeak, the official language of the state Oceania whose enforcement was part of totalitarian politics. Even though based on English, Newspeak is an artificial language imposed to replace the earlier variant, “Oldspeak.” It was expected that Newspeak would ultimately be the only official means of communication. The goal of Newspeak was to express the ideology and worldview of “English Socialism” (Ingsoc) and to control the way that citizens thought. As Orwell wrote, “it was intended that when Newspeak had been adopted once and for all and Oldspeak forgotten, a heretical thought—that is, a thought diverging from the principles of Ingsoc—should be literally unthinkable, at least so far as thought is dependent on words” (1949, pp. 328–329). As he further explained:

This was done partly by the invention of new words, but chiefly by eliminating undesirable words and by stripping such words as remained of unorthodox meanings, and so far as possible of all secondary meanings whatever. To give a single example. The word *free* still existed in Newspeak, but it could only be used in such statements as ‘This dog is free from lice’ or ‘This field is free from weeds.’ It could not be used in its old sense of ‘politically free’ or ‘intellectually free’ since political and intellectual freedom no longer existed even as concepts and were therefore of necessity nameless. (Orwell 1949, p. 329)

The Orwellian concept of Newspeak has been adopted by linguists, including the Polish professor of language theory Michał Głowiński (1934b), who analyzed the official language of the People’s Republic of Poland. Newspeak was also a focus of study of scholars in Czechoslovakia. Both the philologist Karel Palek, alias Petr Fidelius (1948b), and the dissident author and politician Václav Havel (1936–2011) wrote about how language in totalitarian societies can be used as a tool designed to control thought (Tuckerová 2010). According to the analyses of Głowiński, Fidelius, and Havel, Newspeak was an aggressive social language that tended to usurp other linguistic areas and types of social languages. The supremacy of Newspeak was rooted in the political, economic, and physical power held by politicians and functionaries of the totalitarian state over citizens. The ideological sources of Newspeak mainly drew on Marxist philosophy and works of Vladimir Lenin.

Newspeak, in terms of language theory, has been seen as an official and formal type of communication used by political authorities at every level of power: national, regional, and local (Głowiński 2009a, pp. 53–58). It was applied by every institution that belonged to, or was controlled by, communist authorities, including the media, state agencies, factories and other workplaces, and schools and universities. It is in this sense that Newspeak can be viewed as a language of power. Newspeak was invented by the authorities in Poland, Czechoslovakia, and other countries, and hence imposed on Polish, Czech, and other native languages. However, Newspeak transformed those native languages by destroying their natural features. It was impossible, for example, to communicate feelings and ideas in Newspeak. Its main purpose was to eliminate spontaneous communication between people, and to confirm communist ideology and communist power as the only possible reality.

As pointed out by pointed out by Havel, Fidelius, and Głowiński, the most striking quality of Newspeak was its extreme schematization. The relationship between a word and an idea was not only stable but also static and unchangeable. Some words and ideas were sharply limited to a particular context. Due to this process, the language of communism became inflexible. It is notable that this kind of pattern fits the general structures of slogans used in advertising and propaganda; however, in the case of communist language, the whole process of communication was subordinated to the same rule.

Such schematization overwhelmed all acts of communication and led to a loss of authenticity in the language. Language reflects intersubjective beliefs and feelings shared by a community that words and sentences have a reference to reality; in communist societies, language users distrusted Newspeak because they felt that its chosen schematization did not have any relevance to their social reality. Havel termed the lack of reference between Newspeak and the meaningful social reality “verbal mysticism” and “evasive thinking.” Głowiński called it “ritualization of language.” Instead of expressing and creating the social reality, Newspeak language functioned somewhat like the huge banners bearing communist slogans about peace, happiness, and order, which covered the dirt and mud of city centers across East and Central Europe. As Havel pointed out: “From being a means of signifying reality, and of enabling us to come to an understanding of it, language seems to have become an end in itself” (1992, p. 12).

Havel was not the only one who applied Newspeak as a motif to portray emptiness, inauthenticity, and lack of social relevance. It was also frequently employed by writers, playwrights, and filmmakers in the anti-totalitarian underground culture. Newspeak was perceived as artificial, empty, and alien. It indicated the separation between the society and the official language of political power. In the poem “The Power of Taste,” the Polish poet Zbigniew Herbert described the duality between society and Newspeak:

Verily their rhetoric was made of cheap sacking

(Marcus Tullius kept turning in his grave)

chains of tautologies a couple of concepts like flails

the dialectics of slaughterers no distinctions in reasoning

syntax deprived of beauty of the subjunctive. (1987)

Further, Petr Fidelius showed that propaganda in the totalitarian state is different from that of the revolutionary one. Revolutionaries, Fidelius argued, are people of the idea. They want to persuade (or force) others to their point of view, and they use language and propaganda to change people’s opinions. When a state becomes totalitarian, language and propaganda alter their nature: the goal is no longer persuasion but rather maintaining power by limiting people’s ability to think independently (Fidelis 1998, pp. 180–182). Language, turned into a ritualistic code, makes it impossible to create any new association or reference between word, notion, and reality.

In totalitarian countries, all social and even individual activity is controlled by the state. Eradicating alternative social languages was both a goal and a result of political restrictions. In a totalitarian state there is only one source of power, one voice. The success of Newspeak explains why the communist powers were so active in banning, or at least in limiting, independent voices like the television or radio broadcasts, such as Radio Free Europe, from capitalist countries.

The ideological implications of Newspeak easily expanded from politics to every other aspect of social life. Political and ideological interpretations were present in philosophy, history, philology, archeology, and popular culture (Bednarek 1997). Moreover, some disciplines, like sociology, were banned during Stalinism as being politically incorrect. Unsurprisingly, science was not spared from Newspeak.

According to Głowiński, the first distinguishing feature of Newspeak is strong valuation and binary oppositions. Newspeak does not describe reality in a neutral sense. Rather, it always contains a clear and strong contrasting evaluation. Moreover, the semantic meaning is secondary to the valuation. The semantic meaning can be unclear, but the evaluation must be plain (Głowiński 2009b, p. 12). Newspeak is based on a clear and constant dichotomy, such as ours/theirs, progressive/regressive, or friend/enemy. Głowiński noted that Newspeak, much more than other kinds of social languages, is monovalent, having an inner and immanent axiology. Newspeak indicates and imposes only one possible set of values. For example, the expression “power of progress” (“siły postępu”) connotes nothing more and nothing less than socialism and its supporters.

The second feature of Newspeak is a ritualization of language (Głowiński 2009b, p. 13). Verbal communication is conservative; the vocabulary and structure of sentences are limited and imposed by the context of political events, actions, and circumstances. Always using the same expressions and repeating the same verbal structures and linguistic stereotypes were essential features of the communist language. For example, the expression “party and government” (“partia i rząd”) used to be the only possible linguistic option; the reverse order of the expression “government and party” was not possible in Newspeak, because it questions the leading role of the party in the totalitarian country. Spontaneous acts of verbal expression or spontaneous communication were forbidden. Linguistic rituals were strictly connected with communist holidays and the quasi-religious communist calendar (Holiday of October Revolution, Workers’ Day, Women’s Day, etc.) (Strządała 2003).

A third feature is the magical function of language. Newspeak is an extremely practical language. Statements have a strong, direct influence on people’s behavior. Words in Newspeak do not describe reality, they create it. Talking about wishes as if they were the reality is one of the most striking attributes of Newspeak (Głowiński 2009b, pp. 13–14). At the same time, Newspeak offers a magical annihilation—that is, avoiding and rejecting some terms, word, concepts, and names that are sentenced to nonexistence, like unperson from Orwell’s *1984*.

Other typical qualities of Newspeak are the militarization of language, frequent use of neologisms and euphemisms, and an enigmatic style when dealing with unwanted or unpleasant information (Głowiński 2009b, pp. 2–24). An example of this is using the expression “temporary difficulties” instead of “economic crisis” and “price correction” instead of “inflation” (Głowiński 2009a, pp. 34–43). Moreover, according to Głowiński, Newspeak is based on arbitrary decisions and the will of the authorities. Routine censorship was one obvious example of totalitarian practice reflected in Newspeak (Głowiński 2009b, p. 14). Newspeak is imposed by political power. It is the antipode to freedom of speech.

## Newspeak in the New Biology

The New Biology was imposed in Poland in 1948 following the consolidation of the communists by the Polish United Workers Party (PZPR) and the totalitarian shift in the country. Monopolization of power is, historically speaking, strictly connected with the elimination of independent voices (Arendt 1993). That is the reason why other alternative sources of authority—like universities, scientific societies, public media, the Roman Catholic Church, and other traditional public institutions–were targeted by aggressive censorship policies. This expansion of communist political power and Marxist-Leninist ideology occurred not only in a strictly political arena, but also in a linguistic dimension. Newspeak tended to be a universal, expansionist language that dominated every other social language and all public discourse. Newspeak operated in the language of science as well, and Lysenkoism is the example of this phenomenon in biology.

The New Biology in Polish zoology exhibits all the rules of Newspeak. First, we find clear valuation and binary oppositions. Newspeak in the case of zoology is based on simple dichotomies such as “Michurinism”^6^ versus “Morganism,” “Creative Darwinism” (“Twórczy Darwinizm”) versus “Darwinism” (“Darwinizm”), and “New Biology” versus “old biology” (“stara biologia”). The first element of each dyad is positive and connotes progress and political correctness, while the second element indicates political enemies. Genetics was always referred to as a “bourgeois, criminal science,” while the New Biology was called a “progressive, truly materialistic science” (Michajłow 1949, pp. 88–89; Olbrycht 1949, pp. 99–107).

Typical of Newspeak is the frequent use of neologisms such as “Creative Darwinism,” “creative substance,” “stadial development,” etc. The semantic meaning can blur, but the value judgement these phrases contained was clear. For example, the New Biology adopted the concept of “creative substance,” introduced by Olga Lepieszynska, and rejected cell theory. However, no one provided any definition or description of the “creative substance” she had supposedly discovered. Instead, the theory of “creative substance” was reported as “progressive,” “revolutionary,” and announced as a “breakthrough” in science (Raabe 1953, pp. 42–46; Skowron 1953). Even the very basic question of whether or not the creative substance exists was completely irrelevant. No one dared to publicly demand scientific evidence or proof.

During the conference proclaiming the New Biology in Poland, held in Warsaw in March 1949, one of the leaders of Lysenkoism in zoology, Włodzimierz Michajłow, said: “The powers of progress are on Łysenko’s side, the powers of reactionists are against him” (1949, p. 87). This sentence is an example of simple and sharp binary oppositions, on the one hand, and of inner axiology on the other. Reactionaries who are political enemies are identified with the researchers rejecting the theories of Lysenko and his followers. Political correctness is associated with those who support Lysenko’s ideas. Strong valuation is essential to Michajłow’s statement.

The essential feature of any scientific publication is originality, and plagiarism is, of course, unacceptable. By contrast, the distinguishing feature of Lysenkoist publications is the constant repetition of the same topics, using the same vocabulary and making reference to the same publications. For a Lysenkoist publication, the less original, the better. The original papers that were based on real research were subject to censorship; for example, Jerzy Konorsky’s original research on neurobiology was censored (Ber 1956, p. 5) because his work was incompatible with Pavlovism (Jaczewski 1951, p. 240). Newspeak ritualized repetitive language and eliminated scientific creativity.

With regard to the magical function of language, extravagant claims were made for the introduction of the New Biology in zoology. Special Lysenkoist techniques for breeding cows, chickens, sheep, and pigs were expected to benefit a society due to the inheritance of acquired characteristics (Michajłow 1952). For example, exposing animals to colder temperatures was supposed to make them strong, healthy, and more productive and thus improve the quality of their meat (Pająk 1949, pp. 82–86). None of these ideas were proven, yet they were applied in some State Agricultural Farms, which was one of the factors along with nationalization of trade, collectivization of land, and restriction of private farming that led to food shortages in the country (Kalinski 1995, pp. 9–47).

Another example of New Biology magical thinking is polyspermy (see Strządała 2012b). Polyspermy means the fertilization of an egg by more than one sperm, which in the case of mammals almost never happens in nature or is lethal for the embryo. Lysenkoists claimed that polyspermy not only occurs in mammals, but has a positive influence on the biological quality of offspring. They said it should be used with cows and pigs to improve their meat (Pająk 1949, p. 90; Skowron and Fidelus 1954). There were many Lysenkoist publications describing polyspermy and its positive outcomes, two of them written by a former specialist in genetics, Marchlewski (1951a, 1954). Lysenkoist publications described virtually non-existent biological phenomena as though they had been implemented in practice and really worked.

Any scientist who openly rejected Newspeak was forced into silence by being prohibited from having any contact with students or not being allowed to publish their works. For example, Konorski’s articles were blocked through internal scientific censorship (Ber 1956), and Edmund Malinowski, the former pioneer of Polish genetics, was ostracized at the 1950 Lysenko conference in Kuźnice, despite his conversion to the New Biology, because his paper used the forbidden term “mutation” when discussing speciation (Malinowski 1951). Afterward Malinowski was publicly humiliated and treated as an “unperson,” cast out from the scientific community (Jaczewski 1951).

The fourth hallmark of Newspeak is that language is based on arbitrary political decisions. In many zoological publications, Stalin and his works were referred to as authoritative. For example, in the Polish biological journal *Kosmos* in 1953, Stalin was admired for his significant influence on biology. His support for Lysenko, Michurin, and Lepieszynska is treated as proof that their theories are correct (Anonymous 1953, pp. 3–5).

Finally, Newspeak features the militarization of language. The introduction of the New Biology in zoology was frequently portrayed as a battle, war, or conflict between its followers and the supporters of genetics. While analyzing the Lysenko conference in Dziwnów in 1952, for example, Adam Drozdowicz wrote that “taking the side of Creative Darwinism has been and continues to be a fierce battle in the class struggle” (1953, p. 72). Articles and public presentations about the New Biology were full of militaristic metaphors. For example, Michajłow said: “The Soviet researcher is not a loner working for his own pleasure. He is a commander of a certain unit of the fight, a fight for the well-being and progress of the people” (1949, p. 92). The New Biology, according to Michajłow, leads directly to the liberation of the working class (1949, p. 89).

## New Biology: A Scientific Theory or a Verbal Ritual?

Scientific language is a kind of professional jargon unique among other linguistic genres. The language of science is highly specialized, consisting of technical vocabulary, which usually cannot be understood by a person who is not educated in the particular field being discussed. According to Charles Bazerman, a specialist in the rhetoric of science, scientific language is “distinct from our everyday conversation and newspaper reading” (1988, p. 293). Conventional speech is frequently blurry and imprecise because one of the functions of natural language is not only informing but also expressing emotions. Scientific language is based on clear terms and definitions. Scientific procedures need to be described in detail in order to be repeated, confirmed, or questioned by further research. The New Biology lacked these basic features of scientific language. It was full of ideas and terms that had no precise definitions. For example, what might “stadial development” or “polyspermy” mean in terms that applied to mammals? Lysenkoists claimed that these methods should be employed in breeding cows, sheep, or pigs, but how could they be used if they were never explained or specified in the case of mammals?

The use of scientific language is a condition for participating in the scientific community, and this social aspect of scientific language is sometimes seen as a weakness. The social control, special practices, and rules that emerge among researchers who form a specific social group are perceived as subjective and contrary to the procedures of objectivity. However, truth, facts, and knowledge always have social aspects and are socially constructed (see Berger and Luckman 1966). This should not necessarily be seen as problematic because the most objective truth, which somehow “occurs” outside of human societies, is irrelevant logically and cognitively, as well as emotionally and ethically, to human life.

Bazerman points out, following other philosophers and scientists before him–from Kant’s *a priory* and *a posteriori* statements (2010 [1787]) to Fleck’s concept of epistemological collectivism (1986 [1935]) 1986—that cognitive processes are not objective but rather incorporate assumptions about the nature of reality, as well as ideological components from outside the realm of science. What is more, scientific language plays certain social functions, such as establishing and maintaining the authority of science. It “serves the competitive interests of separate individuals and research groups” and is “often fuzzy, incomplete, undefined” (Bazerman 1988, p. 294). The question is, was the Newspeak in New Biology just another kind of scientific language, with all its imperfections, or was it a significantly different phenomenon?

I suggest that the language of the New Biology was definitely a semiotic system: it incorporated assumptions about reality and ontological statements, and it incorporated the ideology of Marxism and Leninism. For example, one of its claims was the rejection of the idealism that was identified with Western science, and particularly genetics (Michajłow 1953, pp. 10–24). The New Biology rejected the concept of genes and chromosomes (Dembowski 1949, pp. 55–56). However, one of the principles of modern science is that statements must be verified experimentally (Popper 1992). When Lysenkoists were asked during the conference in Kuźnice about evidence for claims made under the banner of the New Biology, one Lysenkoist, Jan Pająk, answered flatly that there were already plenty of articles on this topic, and there was no need to check or verify them (Anonymous 1951, pp. 278–279).

The main historical issue here, then, is not that the New Biology was based on materialism and genetics on idealism, but rather that it contained ironic inconsistency. Genetics was accused of idealism even though it offered proof of the material existence of genes. The materialistic New Biology was based on ideas, but in the case of zoology these had no reference to reality in this field of knowledge. Converting to the New Biology demanded not a classic “paradigm shift” (Kuhn 1962 [2001]), but an adaptation to the “new reality,” which required scientists, at least publicly, to be in accordance with the ideology of Marxism and Leninism (Olbrycht 1949, pp. 102–103). For example, claims that inborn qualities (genes) do not determine the nature of living things but are dependent on environmental factors and conditions (Dembowski 1949, pp. 49–55) were compatible with Leninism. Therefore, by changing the conditions of existence, Lysenkoists claimed it was possible to alter nature. The main assumption of the New Biology was that nature can be changed. Thus, the New Biology’s ideals did not require proof of any theories or hypotheses. It claimed, as Dembowski put it, to create both new facts and new biological phenomena (1949, pp. 54–55).

The relationship to reality is the biggest difference between Darwinism, which describes the rules of evolution, and Creative Darwinism, which generates new nature (Dembowski 1949, p. 15). Creative Darwinism does not describe the past. Its goal is to take control over nature in order to use the results in social practice (Michajłow 1953, pp. 10–24). Hence, the New Biology was supported by new theories and proved hypotheses that were meant to be realized in practice. Practice was more important than evidence or theories. This is a reversal of the hierarchy of modern science, where practice (technology, farming, agriculture) is a result of applied science. In the New Biology, in short, practice was the singular aim of academic effort. As one of Lysenko’s followers put it, “Michurin science is based on the principles of materialistic dialectic and it is attempting to transform nature in the interest of the people” (Jeżikow 1952, p. 86). In this context, asking for evidence is superfluous. Facts were not to be described or negotiated, but rather invented.

Bazerman argues that scientific language serves to establish or maintain the authority of science, mainly by exclusion and intimidation. The New Biology definitely used this linguistic tool to impose Lysenko’s ideas on Polish zoology. However, it is doubtful that the language of the New Biology was used in order to maintain the authority of science. On the contrary, it was a systematic project for establishing the ideology of Marxism and Leninism in every aspect of social life, including the scientific milieu, by destroying the authority of science itself (Michajłow 1953, pp. 10–24).

Newspeak in biology not only consisted of ideological components, but of claims that were more important than the biological phenomena themselves. As Kazimierz Petrusewicz, one of the foremost proponents of Lysenkoism in Poland, put it, “The frontline of the ideological war marches hrough the natural science” (1951, p. 1). Ideological components of the New Biology were seen as self-evident advantages, while criticism of the lack of evidence to prove that Lysenko’s ideas actually worked in animal breeding was treated exclusively as a political attack (Olbrycht 1949, pp. 99–101).

Presentations and publications in the New Biology are more reminiscent of verbal ritual than a well-constructed scientific argument. Rituals can be performed by repeating certain actions (gestures, movements of the body, words, texts, etc.) in a certain sequence and order. The inalterability of the elements guarantees their validity. Rituals, as linquistic functionalists and structuralists note, are a mechanism to introduce, reestablish, or maintain the social order, to enforce systems of values, and to confirm authorities symbolically (see van Gennep 2006; Leach (1976) [2010]; Geertz 1973). Rituals not only stabilize social roles and social reality, but shape it too.

The same was true in the case of the New Biology, in which all kinds of performances (speeches, articles, and conferences) were not a form of communicating or consulting original scientific findings, but were rather used as a means to reshape a political order and to change the structure of the scientific community by establishing authority based on political order. The Lysenkoist texts were full of particular expressions, linguistic schemes, names, and terms that were repeated over and over without revealing anything new that provided additional evidence (or *any* evidence, for that matter) of claims previously made.

Schematization was one of the most striking features of the language of New Biology. Furthermore, inner axiology and strong judgments incorporated in the language of the New Biology were tools of enforcing a new system of values. Brought together with an imposed Soviet ideology, Newspeak, rather than communicating novel ideas, confirmed the ritualistic (not the scientific) nature of Lysenko’s ideas used in Polish biology.

## Naming New Biology: From Michurinism to Lysenkoism

The linguistic changes that took place in Polish zoology coincided with the rise and fall of Stalinism in Poland (1948–1956). Political shifts shaped the renaming of new politically correct ideas in biology. Before the introduction of Stalinism, some of Michurin’s ideas were known, but they not widely accepted in Poland. Prior to 1948, Polish scholars sometimes used the term “Michurinism” (“Miczurinizm”) to denote the conviction that acquired characteristics could be inherited. This meaning was shared by both the minority who supported Michurin’s hypotheses, such as Jan Dembowski, as well as the majority of Polish scientists, who rejected or ignored Michurinism due to its inconsistency with genetics (Dembowski 1927). In 1948, the prevalent expression Polish zoologists used to describe the new politically proper trends became “theories of Michurin and Lysenko” (“teorie Miczurina-Łysenki”). The surname of Lysenko was added to Michurin. Between 1949 and 1953, “Creative Darwinism,” “New Biology,” and “New Genetics” became the dominant terms, connoting, in the case of Polish zoology, concepts such as polyspermy as enhancing the vitality of animals (Bilewicz and Kamiński 1952), the regeneration of animals (Skowron 1951, 1952, pp. 3–10), and stadial development (Michajłow 1952), along with the reinterpretation of the Darwinian concept of speciation and the inheritance of acquired characteristics.

Some of these concepts were not just simply copied from Trofim Lysenko’s works but were enriched by his Polish followers (such as Skowron and Michajłow), whose aim was to transfer some of Lysenko’s ideas—like vegetative reproduction (asexual reproduction occurring in plants)—into the realm of mammals and birds (see Strządała 2012b). Hence, in Polish zoology, the New Biology was more of a futuristic project than a description of scientific findings, since none of those ideas (vegetative reproduction, polyspermy, regeneration of mammals, etc.) were ever observed or described but merely postulated.

Supporters of Lysenko frequently avoided the term “genetics” and instead used the term “Mendelism-Morganism,” sometimes adding in “Weismannism” for good measure. Avoiding the term genetics served two purposes. One was the magical rhetorical annihilation of genetics as a concept and a science. It was unacceptable, as Malinowski discovered at the Kuźnice conference, to use terms like “mutation” and “genes,” even if one was being critical of genetics. The second purpose was to identify the whole science of genetics with particular researchers, like Gregor Mendel, Thomas Hunt Morgan, or August Weismann, in order to diminish the broader scientific significance of genetics and to question its objectivity. Genetics as “Mendelism-Weismannism-Morganism” was not a branch of biology or even a science, for that matter. It was simply the subjective beliefs of three men, whose biographical features (i.e., Mendel was a priest, Weismann carried out brutal experiments on mice, etc.) could also be cited as “proof” of why their beliefs were wrong.

Furthermore, a term coined from a surname can often have a negative emotional connotation in the Polish language—and thus can signal a political shift. For example, the same pattern was used in the case of “Hitlerism” and later “Stalinism.” Some expressions like “Darwinism,” to convey the theory of evolution, have neutral or positive connotations; however, in Polish the implication is typically negative. This explains why, at the height of the Lysenko period in Poland, his supporters never attached the suffix “ism” to his name when referring to his theories but rather used terms like the New Biology. When the term *Lysenkoism* (“Łysenkizm”) was finally invoked in 1955, it signified its namesake’s downfall.

The same process can be seen in the case of the ideologization of neurophysiology. Discoveries by the Russian researcher Ivan Pavlov about conditioned responses and reflex actions, whose scientific and historical significance were not questioned, were used by the communist system to justify its ideological claims. A special month-long conference dedicated to the reinterpretation of Pavlov’s works was organized in Krynica, Poland in December and January 1952 (Anonymous 1952). The ideological interpretation of Pavlov’s classical conditioning stated that a conditional response can be turned into an inherited automatic reflex, thanks to the inheritance of acquired characteristics, one of the most important elements of New Biology. In turn, New Biology could be used to create a new communist society and a new communist human being. When Lysenko’s theories were being rejected by Polish biologists in 1956, the term “Pavlovism” (“Pawłowizm”) was coined. In this case, however, the term was coined not as a reference to Pavlov’s findings, but to the mechanism of social pressure (coercion and compliance) used by Lysenko’s followers in the scientific community to condition politically proper reactions (Ber 1956, pp. 1–5).

New Biology was a general term that bound together the theories of Lysenko with other associated theories, like Lepeshinskaya’s “creative substance,” and was treated as a keyword for a new ideological breakthrough in biology. Creative Darwinism, on the other hand, applied to the Lysenkoist reinterpretation of Darwinism and had the practical aim of shaping nature in accordance with the government policy of central planning.

As mentioned above, the term “Lysenkoism” only appeared in Poland once criticism of Lysenko’s biological ideas began to surface. The first use of this term appeared in an article “Przerwijmy zmowę milczenia. Darwinizm a łysenkizm” (“Let’s break the conspiracy of silence: Lysenkoism and Darwinism”) by a young Polish zoologist named Leszek Kuźnicki (1955). “Lysenkoism” in this sense meant the ideologization of science, limiting the freedom of scientific research, promoting dogmatism instead of scientific debates, and denoting the lack of evidence for the New Biology (Strządała 2012a). The next critique of Lysenko appeared in an article by Jan Bóbr (1956), wherein the term “Lysenkoism” was used to diagnose a pathology in science. “Lysenkoism” meant the way a scientific milieu could be manipulated and utilized to signify political and ideological shifts (Bóbr 1956, pp. 1, 6). By the mid-1950s, Kuźnicki and Bóbr were joined by other voices. Zoologist Władysław Grodziński, for example, pointed out the lack of evidence for the New Biology, the deficiency of logic in Lysenkoist reasoning, as well as the highly doubtful methodological approach of the man and his followers (1956, p. 6).

The criticism of Lysenkoism enabled the rejection of Newspeak. After 1955, the New Biology was renamed Lysenkoism. As a result, “Lysenkoism” is used to denote a historically specific ideological and political turn in Polish biology. It does not refer to Lysenko’s theories, but rather to the attempt to transform zoology in accordance with Lysenko’s ideas in spite of the obvious problem that animals and plants have fundamental differences. The act of renaming the New Biology as Lysenkoism was thus a rite of disapproval and rejection of its original claims, which illustrated magical thinking, ritualization, binary oppositions, and, ultimately, the symbolic power of this specific language framing. In short, renaming the New Biology as Lysenkoism was an act of public condemnation.

## Conclusion

Newspeak is a type of language that continues to be an essential element of propaganda in totalitarian countries, but it also influenced the lingo of democratic or capitalist societies in areas such as advertising. The main purpose of Newspeak is not communication between individuals, but creating a new order, eliminating freedom of speech, and imposing a certain way of thinking by destroying spontaneous acts of verbal communication and dictating an ideological dictionary. As the example of Lysenkoism and the New Biology shows, the tyranny of Newspeak can extend even to the supposedly neutral and objective language of science.

The language surrounding the New Biology meets all the rules of Newspeak. At the linguistic level, the New Biology in Poland was distinguished from language used in biology before, after, and outside of Lysenkoist discourse. The language of the New Biology in Poland consisted of warrior metaphors, strong valuations, schematizations, and direct political references. What is more, it declared the importance of producing certain “findings” rather than informing about new discoveries. These “findings” did not arise as the products of experiment—proving or disproving some hypothesis or predicted theory. Instead, they were designed in advance and compulsory. While presenting the results in conferences or in articles, research procedures and methodology were blurred or omitted. As a consequence, experiments were diminished and could not be repeated, confirmed, or disproved.

Producing New Biology “findings” was not the result of scientific research; it was a political duty. The New Biology functioned to “manufacture” biological phenomena. It was a tautological system lacking any link to reality outside its political and ideological boundaries. I identified the language of this system as Newspeak, a concept previously applied to other areas of communist culture such as art, music, the press, and official media. The New Biology and its manifestations represented a closed and coherent system of concepts, more reminiscent of a ritual than a scientific theory. Without performance of these specific ritualistic codes, the New Biology could not be maintained. Abandoning the practice of Newspeak led to the evaporation of the New Biology—like any ritual, the New Biology could not be preserved without repeating a certain code. The New Biology was not rejected due to some new findings that disproved its theories, but because Polish zoologists changed the code in alignment with a shift of power.

## Footnotes

1 In Polish historiography, the years 1948–1956 are considered the Stalinist period that was launched by the takeover of power by Bolesław Bierut (1892–1956) and his supporters. This represented the peak of totalitarian tendencies in Poland and the highest dependency on the authority of the Soviet Union. In the period immediately after the end of World War II until 1948, the terror began and some elements of Stalinism were present but not fully established. Some characteristics of a pluralist society still existed in the public sphere, and science was not yet fully controlled by politics. The year 1948 was a turning point in the process of Stalinization, of which the enforcement of Lysenkoism was part. After 1956, when the regime loosened up, there was still authoritarian rule, but it was no longer Stalinist (see Köhler 2013; Eisler and Kupiecki 1992; Paczkowski 1999; Werblan 2009).

2 Dembowski was a Polish zoologist born and raised in Russia and a communist politician in the Polish People’s Republic. He was the marshal of parliament (1952–1957), the Deputy Chairman of the Polish Council of State (1952–1957), and the first president and co-founder of the Polish Academy of Sciences (1952–1956)..

3 For more about Dembowski’s process of imposing the New Biology in Poland, see Strządała (2011).

4 On the process of introducing the New Biology, see de Jong-Lambert (2008), Köhler (2008), and Strządała (2012b).

5 The Secret Teaching Organization (or Society) was an underground educational society established in occupied Poland (1939–1945) to provide primary, secondary, and higher education to the masses as a response to the German policy of limiting the education of Poles to a few years of elementary school.

6 Ivan Michurin (1855–1935) was a Russian grower and selectionist who worked on the hybridization of distant kinds of plants and introduced new methods of plant cultivation. Michurin developed some previously popular ideas that cold makes plants, animals, and humans stronger and therefore healthier. His contribution to the development of selection and agriculture was taken over by Lysenkoism and incorporated into Soviet anti-genetic propaganda.
